# DOG1-Mediated Priming Followed by Environmentally Tunable Plasticity: A Two-Phase Model for Dormancy Establishment in *Xanthium strumarium*

**DOI:** 10.3390/proteomes14030042

**Published:** 2026-08-21

**Authors:** Iman Nemati, Somayeh Gholizadeh, Dinakaran Elango, Sara Hamzelou, Karthik Shantharam Kamath, Mohammad Sedghi, Reza Tavakkol Afshari, Paul A. Haynes

**Affiliations:** 1 Department of Plant Production and Genetics Engineering, Faculty of Agriculture and Natural Resources, University of Mohaghegh Ardabili, Ardabil 56199-11367, Iran; 2 Department of Plant Breeding and Biotechnology, Faculty of Agriculture, University of Tabriz, Tabriz 51666-16471, Iran; 3 Department of Agronomy, Iowa State University, Ames, IA 50011, USA; dinakaranelango@gmail.com; 4 School of Natural Sciences, Macquarie University, North Ryde, NSW 2109, Australiakarthik.kamath@mq.edu.au (K.S.K.); 5 Health and Biosecurity, Commonwealth Scientific and Industrial Research Organization (CSIRO), Adelaide, SA 5000, Australia; 6 Australian Proteome Analysis Facility, Macquarie University, North Ryde, NSW 2109, Australia; 7 Department of Agrotechnology, Faculty of Agriculture, Ferdowsi University of Mashhad, Mashhad 91779-48974, Iran

**Keywords:** *Xanthium strumarium*, seed dormancy, proteomics, metabolic suppression, epigenetic control, ABA, DOG1

## Abstract

Background: Seed dormancy is crucial for plant survival and agricultural productivity, yet its molecular mechanisms, particularly the role of maternal effects, remain poorly understood. Methods: In this study, we applied a SWATH-based, label-free, quantitative shotgun proteomic mass spectrometry approach to investigate the temporal dynamics of dormancy establishment in *Xanthium strumarium*, a wild plant with two seeds in one burr that, despite sharing the same genetic and environmental conditions, exhibit distinct dormancy states. Results: Our data show that dormant seeds undergo coordinated metabolic suppression, marked by a decrease in energy metabolism, cell cycle arrest, and auxin signaling, explaining their smaller size. Simultaneously, dormant seeds exhibit metabolic re-prioritization towards fatty acid desaturation, cell wall modification, and an active epigenetic program stabilized by dormancy-promoting factors alongside a transcriptionally quiescent state in early–mid development. However, in the late developmental stage, molecular signaling pathways showed a recalibration distinguished by changes in seed metabolism (such as carbon–nitrogen reallocation, sulfur assimilation, and GABA production), hormonal fluctuations, and epigenetic regulation. Notably, previously reported high DOG1 transcript abundance, together with the absence of detectable DOG1 protein in the proteomic dataset, suggests that post-transcriptional mechanisms may contribute to DOG1 regulation. Conclusions: Based on these findings and the available literature, we propose a framework whereby dormancy establishment occurs in two phases: an early DOG1-mediated priming phase followed by a temperature-sensitive plasticity phase during seed maturation.

## 1. Introduction

Seeds are the principal determinants of plant distribution in different geographical areas. As a consequence, plants have evolved seed dormancy strategies, allowing them to sprout only when their environment is optimal. However, maintaining an appropriate dormancy depth is crucial. Low dormancy can cause pre-harvest germination, while excessive dormancy may result in uneven germination. Both scenarios negatively impact seed quality and storage potential [1].

Seed dormancy is recognized as a quantitative trait that varies along a continuum of dormancy depth rather than existing as a simple dormant/non-dormant state. This variation determines seed responsiveness to environmental cues and ultimately influences the timing of germination [2]. To date, research on mechanisms underlying the regulation of dormancy has mainly focused on model plants like Arabidopsis and rice [3,4]. Such studies have provided important information on the molecular mechanisms of seed dormancy. The roles of chromatin remodeling [5,6,7], the major dormancy gene Delay of Germination 1 (DOG1) [8,9], and phytohormones such as abscisic acid (ABA) [10,11], gibberellic acid (GA) [12,13], and ethylene (ET) [14,15] in establishing dormancy in Arabidopsis have been well documented. Despite extensive research, many aspects of seed dormancy regulation are still relatively unexplored. One of the main reasons is that a complex web of genetic, environmental, and maternal factors makes seed dormancy one of the most complicated quantitative traits to study [16].

Rather than being controlled by a single pathway, dormancy is increasingly viewed as an emergent property of interacting regulatory networks. DOG1 has emerged as a central component of dormancy control, acting in close association with ABA-dependent pathways and influencing seed sensitivity to germination-promoting signals [2,17,18]. Histone modifications, chromatin-remodeling complexes, and transcriptional repressors modulate the expression of DOG1 and other dormancy-related genes through crosstalk with different phytohormones [5,7,19]. In particular, temperature-dependent interactions among DOG1, ABA-GA signaling, and epigenetic regulators have been shown to contribute to variation in dormancy depth [20,21,22], while maternal environmental conditions can further modify dormancy phenotypes in offspring seeds [23,24]. These findings emphasize the plastic nature of dormancy regulation and suggest that the final dormancy state results from the integration of multiple developmental and environmental inputs. However, how these regulatory networks are coordinated during seed development to determine dormancy depth remains incompletely understood.

*Xanthium strumarium* (common cocklebur) serves as an exceptional model plant for seed dormancy research due to its intrinsic biological characteristics and ecological adaptability. Crucially, it produces dimorphic seeds within a single burr, one of which is typically dormant and the other non-dormant, providing a naturally controlled system for comparative studies of dormancy mechanisms [25,26]. The seed dimorphism allows researchers to investigate the differential expression of pathways associated with seed size and germination differences between dormant and non-dormant seeds. Additionally, *X. strumarium* exhibits complex dormancy mechanisms influenced by environmental cues; its germination requires specific thermal fluctuations (optimal at ~15 °C daily variation) and is modulated by light conditions [27], thus enabling studies on how environmental factors enforce or terminate dormancy.

Previous transcriptomic and proteomic studies in *X. strumarium* have revealed differential expression patterns between dormant and non-dormant seeds, particularly in genes associated with ABA signaling, developmental progression, and stress responses [25]. Increased abundance of DOG1 in dormant seeds was observed at the transcript level [26], consistent with its proposed role in dormancy induction [28]. Proteomic analysis further revealed delayed activation of cell division, enhanced ABA responsiveness, and changes in seed maturation processes [25]. Nevertheless, DOG1 proteins were undetectable at the proteome level. This highlights the complexity of dormancy regulation and suggests the potential involvement of additional regulatory mechanisms beyond transcript abundance alone.

Although these studies have provided important insights into molecular differences between seed types, they primarily represent static comparisons. Consequently, the temporal organization of molecular events associated with dormancy establishment and the developmental transitions underlying dormancy acquisition remain poorly understood.

In this study, we reanalyzed an existing proteomic dataset from *X. strumarium* seeds with a focus on temporally resolved molecular changes across defined developmental stages (3–10, 10–20, 20–30, and 30–45 days after emergence, designated as Phases I–IV, respectively). This enabled us to investigate the developmental dynamics of dormancy-associated pathways and propose an integrated framework for understanding dormancy regulation in *X. strumarium*.

## 2. Materials and Methods

### 2.1. Plant Material, Growth Conditions, and Sampling

F_2_ seeds of *X. strumarium* L. were grown in a controlled growth chamber (20 °C day/16 °C night, 8 h light at 9000 lux) for 4 months and then transferred to a greenhouse under natural spring light conditions (approximate photoperiod of 14 h light) with temperatures ranging from 20 °C at night to a maximum of 35 °C during the day to stimulate the reproductive phase. By using F_2_ seeds, we minimized maternal environmental influences (e.g., field-derived nutrient disparities) that could bias measurements of seed traits (e.g., seed size/weight and dormancy). This approach also reduced environmental heterogeneity among biological replicates, thereby improving comparability in downstream molecular analyses.

Small and large seeds from tagged burrs (marked with colored ribbons to track emergence time) were then sampled at five developmental time points (3, 10, 20, 30, and 45 days after emergence; DAE), representing key developmental stages of seed formation. The seeds were dissected, flash-frozen in liquid nitrogen, and stored at −80 °C. Biological replicates (*n* = 3) were collected for each developmental stage and seed type.

**Figure 1:**
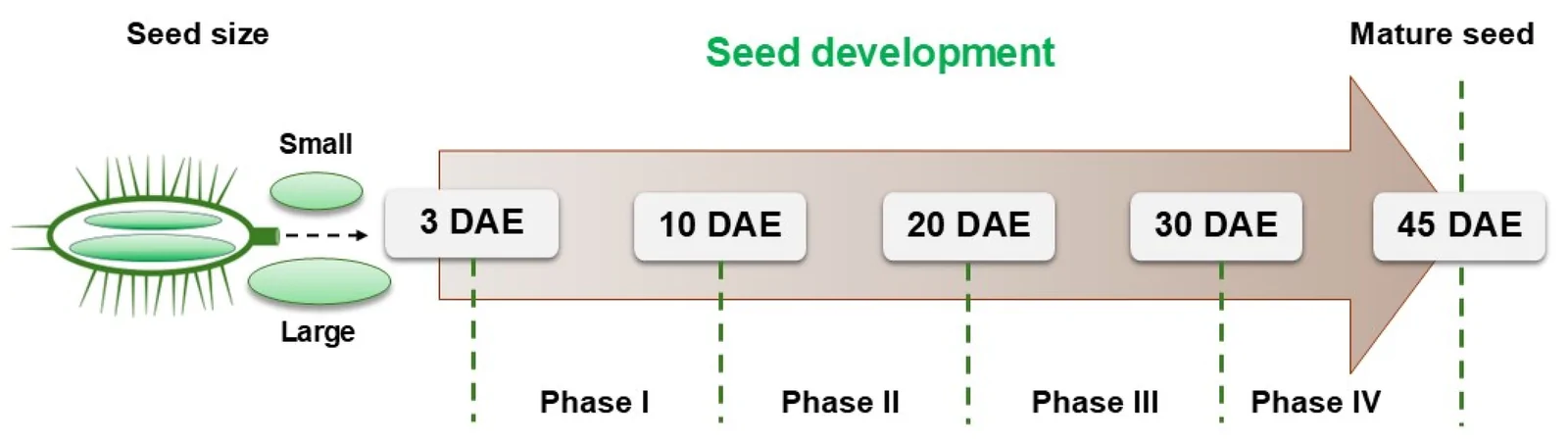
Experimental design and comparative proteomic profiling of seed dormancy in *X. strumarium*. Proteomic data were obtained from five sequential sampling time points (3, 10, 20, 30, and 45 days after burr emergence; DAE). For downstream analysis, the intervals between these points were defined as four distinct physiological phases: Phase I (3–10 DAE), Phase II (10–20 DAE), Phase III (20–30 DAE), and Phase IV (30–45 DAE). Each phase was compared with the subsequent phase in dormant and non-dormant seeds to identify DAPs. This pairwise, phase-sequential comparison strategy enhanced the sensitivity for identifying GO terms associated with specific transitional stages, ultimately facilitating the development of a working hypothesis for dormancy induction.

Since samples were collected independently at each stage, the intervals between these time points were defined as four distinct physiological phases, including Phase I (3–10 DAE), Phase II (10–20 DAE), Phase III (20–30 DAE), and Phase IV (30–45 DAE) (Figure 1). Each phase was compared with the corresponding phase in dormant and non-dormant seeds to identify differentially abundant proteins (DAPs). This approach was used to characterize temporal molecular transitions associated with dormancy development and to improve the detection of phase-specific biological processes and enriched Gene Ontology (GO) terms.

### 2.2. Germination Test and Seed Weight

To evaluate germination differences between small and large seeds, a standardized test was conducted using 400 fully mature seeds (200 per size class) distributed across four replicates (*n* = 4). Seeds were incubated on moist filter paper under controlled germination conditions at 25 °C for 10 days, and germination was recorded daily. Germination percentage was derived as (number of germinated seeds/total seeds tested) × 100. A separate subset of dried mature seeds was utilized for weight evaluation. Seeds from each replicate and size class were counted and weighed to determine the average mass per seed.

Differences in seed mass and germination percentage between dormant and non-dormant seeds were assessed using Student’s *t*-test following normality inspection of data distribution. Statistical significance was accepted at *p* < 0.05.

### 2.3. Proteomic Data Acquisition

#### 2.3.1. Protein Extraction

Proteomic data were obtained from five sequential sampling time points. Protein was extracted from ground seed tissue using TRIzol™ Reagent (Invitrogen, Carlsbad, CA, USA) according to the manufacturer’s instructions, followed by ethanol/isopropanol precipitation and washes.

#### 2.3.2. Tryptic Digestion

Protein samples were lyophilized, then solubilized (100 μL of 8 M urea in 100 mM Tris-HCl, pH 8.0), diluted five-fold with 100 mM Tris-HCl, pH 8.0, reduced (10 mM dithiothreitol, 1 h, room temperature), alkylated (20 mM iodoacetamide, 45 min, room temperature in darkness), and digested with trypsin (overnight at 37 °C, terminated by 1% formic acid). The resulting peptides were then desalted using SDB-RPS stage tips (Sigma-Aldrich, St. Louis, MO, USA), eluted with 200 μL of 80% acetonitrile/5% ammonium hydroxide, vacuum-dried, reconstituted in 0.1% formic acid, and finally quantified via micro-BCA assay (Pierce Biotechnology, Rockford, IL, USA).

#### 2.3.3. Peptide Fractionation

Peptides were fractionated using high-pH reversed-phase chromatography. A pooled aliquot (40 μg) from all samples was diluted in 300 μL of 0.1% trifluoroacetic acid (TFA), followed by fraction collection across an acetonitrile gradient (5%, 7.5%, 10%, 12.5%, 15%, 17.5%, 20%, and 50%). All eight fractions were vacuum-dried and subsequently reconstituted in 0.1% formic acid for downstream analysis.

#### 2.3.4. SWATH-Based Proteomic Profiling

Quantitative proteomic profiling was performed via SWATH LC-MS/MS on a TripleTOF 6600 (Sciex, Redwood City, CA, USA) coupled to an Eksigent nanoLC 400 system with the cHiPLC module, following trap column desalting (Halo-C18 (Advanced Materials Technology, Inc., Wilmington, DE, USA), 160 Å, 2.7 μm, 150 μm × 3.5 cm) using 2% acetonitrile/0.1% formic acid.

#### 2.3.5. Spectral Library Generation

For spectral library generation in IDA mode, peptides were chromatographically separated using an analytical column (Halo-C18, 160 Å, 2.7 μm, 200 μm × 20 cm) with a 60 min linear gradient from 5% to 35% mobile phase B (0.1% formic acid in acetonitrile) at a constant flow rate of 600 nL/min. A time-of-flight mass spectrometry survey scan (*m*/*z* 350–1500, 0.25 s accumulation time) was performed to identify the twenty most intense multiply charged precursor ions (2+ to 4+ charge states, exceeding 200 counts per second). These selected precursors were subsequently subjected to MS/MS analysis with a 100 ms accumulation time across a mass range of *m*/*z* 100–1800, employing a rolling collision energy scheme to optimize fragmentation efficiency. Peptides were then separated using LC conditions identical to those described for IDA analysis [29].

The mass spectrometer was operated in SWATH mode with 100 variable isolation windows (*m*/*z* range 350–1500), dynamically adjusted based on spectral library intensity distributions. MS/MS spectra were acquired with a 40 ms accumulation time per window, employing an optimized rolling collision energy strategy (+10% offset for lower *m*/*z* ranges). Rigorous quality control included randomized injections, blank runs, and consistent nanoflow electrospray ionization (nESI) conditions to minimize batch effects and carryover.

### 2.4. Statistical and Functional Analyses

Protein identification was performed using the Paragon search algorithm implemented in ProteinPilot (V5.0.1; SCIEX, Redwood City, CA, USA). Parameters used included: fixed modification of cysteine with iodoacetamide; enzyme specificity, trypsin with one missed cleavage allowed; search effort set to Thorough ID; instrument set to TripleTOF 6600; and global false discovery rate cutoff of 1%. Biological replicates from each seed type and developmental stage were combined to generate a comprehensive spectral library using the *X. strumarium* in-house transcriptome database previously published with the accession number PRJNA809034 [26]. The constructed library was imported into PeakView Software (V2.2, SCIEX, Redwood City, CA, USA) as a reference ion library for targeted extraction and quantification of SWATH-MS data using the following settings: maximum of 100 peptides per protein and a minimum of 6 transitions per peptide; peptide confidence threshold of 99%; transition false discovery rate (FDR) < 1%; 5 min extraction window; and fragment extraction tolerance of 75 ppm.

Quantitative proteomic data were subjected to preprocessing, normalization, and differential expression analysis employing the DEP package (V4.3.0) implemented in R [30]. Following data structuring with the make_se() function, proteins exhibiting a missing value frequency exceeding 50% were eliminated using the filter_missval() function. To address technical variation arising from between-sample differences, normalization was performed using variance-stabilizing transformation via the normalize_vsn() function. Differential abundance was assessed using linear models combined with empirical Bayes moderation (moderated t-statistic), as executed by the test_diff() function. Adjustment for multiple hypothesis testing was accomplished using the Benjamini–Hochberg FDR correction, implemented through the add_rejections() function. Differentially abundant proteins (DAPs) were defined as those with an adjusted *p*-value below 0.05 and an absolute log_2_ fold-change (FC) of at least 0.5 (corresponding to a 1.5-fold change) between consecutive developmental stages (Supplementary Tables S1 and S2).

The identified proteins were then matched to the previously published transcriptome annotations [26] based on gene/protein annotation identifiers to facilitate functional annotation and biological interpretation.

Principal component analysis (PCA) was performed as an unsupervised exploratory multivariate analysis to visualize the overall variance structure of the proteomic dataset using the plot_pca() function via the DEP package [30]. To further validate the developmental stage-dependent clustering of samples, we also performed canonical variate analysis (CVA) using the lda() function from the MASS package (V7.3.60) in R [31], with developmental stage as the grouping factor. The CVA results were compared with the PCA outcome to assess consistency in sample clustering patterns.

Gene Ontology (GO) enrichment analyses were performed at three complementary levels to capture both global and phase-specific biological patterns during dormancy development. First, GO analysis was conducted using all identified DAPs across both seed types to characterize the overall functional landscape associated with dormancy-related developmental transitions (Supplementary Tables S3 and S4). Second, GO enrichment was independently performed using all identified DAPs within each seed type (dormant and non-dormant seeds) to identify seed-type-specific biological processes and molecular pathways (Supplementary Tables S5 and S6). Third, phase-specific GO analyses were conducted separately for over-represented and under-represented proteins at each developmental phase to identify directional functional changes associated with specific developmental transitions (Supplementary Table S7). Functional annotation and GO enrichment analysis were performed using the hypergeometric test, with FDR-adjusted *p*-values < 0.05 considered statistically significant.

For GO analysis, the data were loaded into the STRING database (https://string-db.org/) [32]. Pathway enrichment analysis was subsequently conducted using the following pipeline: (1) gene set definitions were retrieved from the g:Profiler database (https://biit.cs.ut.ee/gprofiler/gost, accessed on 16 May 2025); (2) RNK files were generated using FC values, with positive and negative values indicating proteins increased and decreased in abundance, respectively; (3) data were processed in GSEA (V4.1.0) for enrichment analysis; and (4) results were imported into Cytoscape (V3.10.1) [33] for network visualization and functional annotation using the EnrichmentMap [34] and AutoAnnotate plugins (Cytoscape software, v3.10.3). EnrichmentMap generated pathway networks based on statistical significance, while AutoAnnotate clustered functionally related pathways.

Data visualization was performed using graphical functions implemented in the ggplot2 [35], GOplot [36], and circlize [37] packages in R. Microsoft Excel (2019) was used for preparing the seed germination and seed weight plots and organizing supplementary GO annotation tables.

To assess the theoretical detectability of DOG1 by SWATH-MS, an in silico tryptic digestion of this protein sequence was performed using the ExPASy PeptideMass tool (https://web.expasy.org/peptide_mass, accessed on 19 June 2026), with the following parameters: enzyme = trypsin (cleavage after K/R, unless followed by P), maximum missed cleavages = 1, peptide mass range = 600–6000 Da, and methionine oxidation as a variable modification. The full-length DOG1 protein sequence (160 aa) from *X. strumarium* was derived from previously published transcriptomic data [26] by in silico translation of the assembled DOG1 transcript. The resulting theoretical peptides are listed in Supplementary Table S8.

## 3. Results

### 3.1. Assessment of Seed Phenotypic Traits

**Figure 2:**
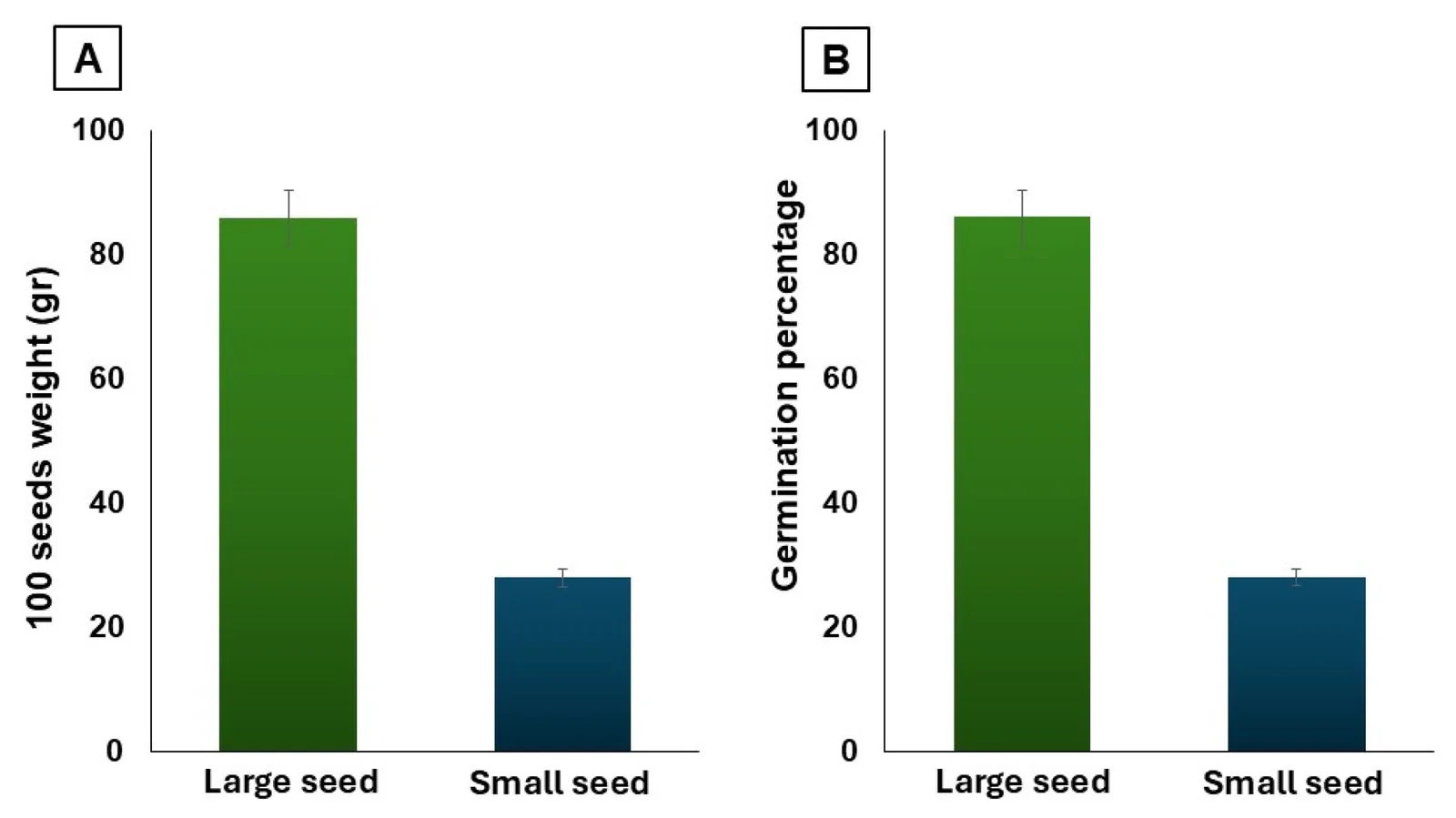
Seed trait variation in *X. strumarium*. (**A**) Mass of individual seeds within a single burr (mean ± SE; *n* = 50; *n* = 4), grouped by size class (large vs. small). (**B**) The differential germination capacity resulting from seed size heteromorphism. Large seeds exhibited a significantly higher germination rate than small seeds after 7 days under identical growth conditions (mean ± SE; *n* = 50; *n* = 4). Statistical significance was determined using Student’s *t*-test (*p* < 0.05).

Comparative analysis revealed significant dimorphism in both seed weight and germination percentage (Figure 2A,B). Large seeds occupying the lower burr position exhibited consistently greater mass and higher germination rates compared to their upper-positioned counterparts. The much greater mass and ≈60% higher germination rate confirm pronounced physiological divergence between the two seeds.

### 3.2. Proteomic Dynamics During Seed Development

**Figure 3:**
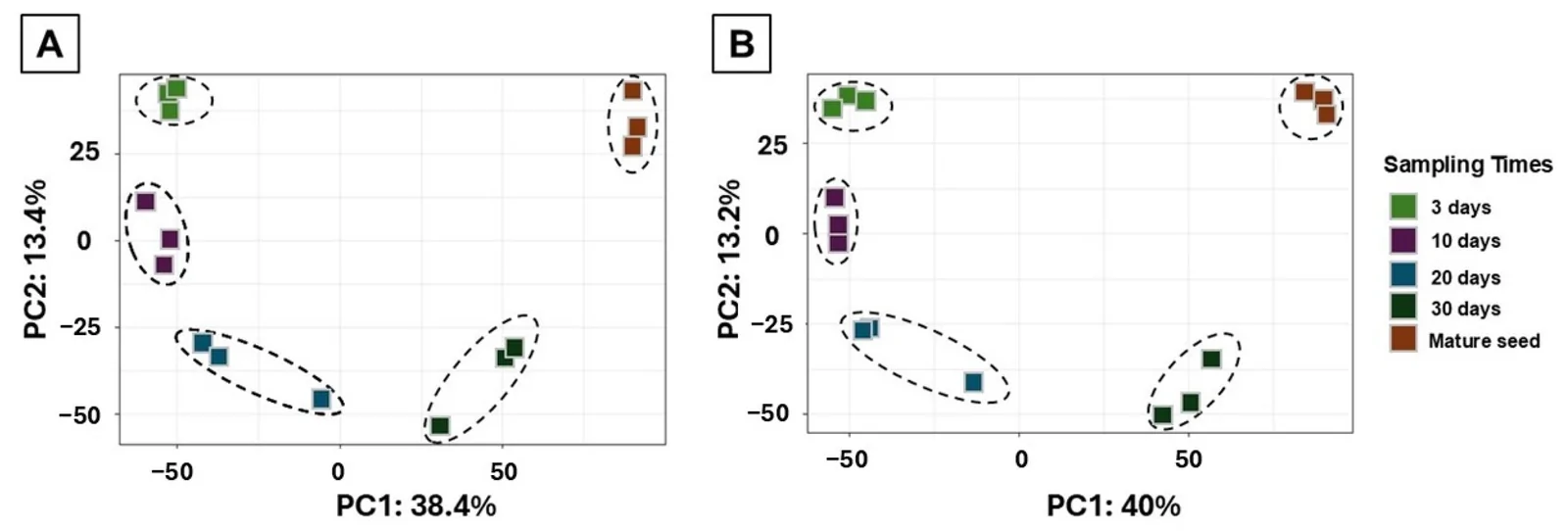
Unsupervised principal component analysis (PCA) of the global proteomic profiles of *X. strumarium* during seed development at 3, 10, 20, 30, and 45 days after burr emergence (DAE). (**A**) Non-dormant seeds (*n* = 3) showing 38.4% variance explained by PC1 (primary separation axis) and 13.4% by PC2. (**B**) Dormant seeds (*n* = 3) with PC1 and PC2 explaining 40.0% and 13.2% of the total variance, respectively. PCA was used as an exploratory approach to visualize temporal shifts in proteomic profiles among developmental stages and seed types.

Principal component analysis (PCA) of the 6524 detected proteins revealed clear separation of samples according to developmental stage in both dormant and non-dormant seeds (Figure 3A,B). Biological replicates clustered closely within each developmental stage, indicating good reproducibility of the proteomic dataset. In addition to the dominant effect of developmental stage, dormant and non-dormant samples exhibited a modest separation, suggesting subtle differences in their global proteomic profiles. Canonical Variate Analysis (CVA), performed as an additional supervised multivariate analysis, confirmed the stage-specific clustering pattern observed by PCA and further demonstrated the close grouping of biological replicates within each developmental stage (Supplementary Figure S1), supporting the robustness of the observed developmental stage-specific proteomic structure.

**Figure 4:**
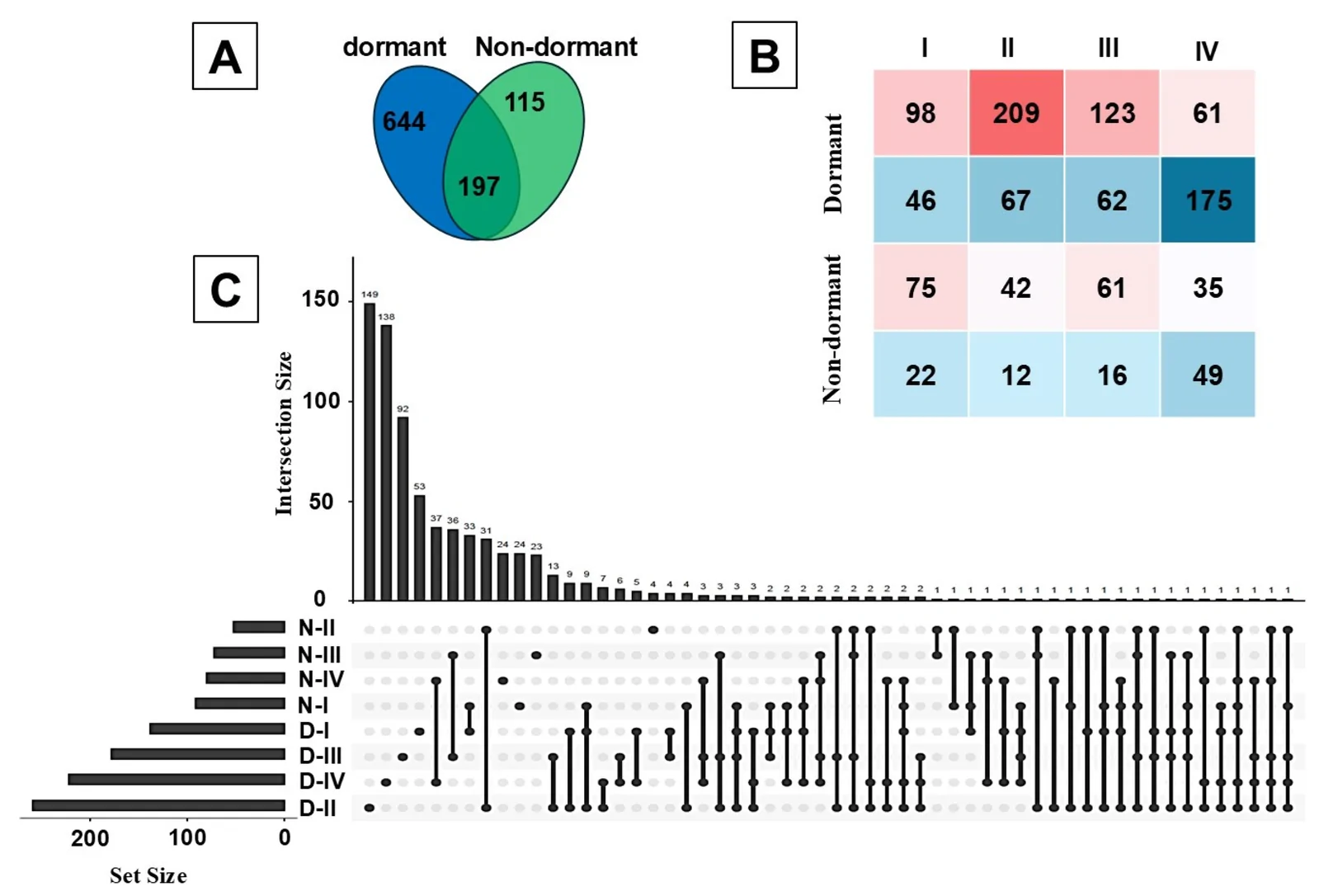
Quantitative proteomic profiling of dormant and non-dormant seeds. (**A**) Proteome overlap: Venn diagram quantifying unique and shared DAPs between dormancy states (dormant-only = blue; non-dormant-only = green; shared = bluish green). (**B**) Number of DAPs (FDR < 0.05, |log_2_FC| > 1.5) from dormant and non-dormant seeds at different developmental phases (I–IV), separated into proteins increased in abundance (red) and decreased in abundance (blue) per phase. (**C**) Phase-specific conservation: Upset plot showing common DAPs between dormant (D) and non-dormant (N) seeds across phases (I–IV).

Comparative proteomic analysis further revealed distinct abundance profiles between seed types across developmental phases. In total, 1173 DAPs were identified across eight comparisons (four developmental phases in two seed types). Non-dormant seeds contained 312 DAPs, while dormant seeds exhibited nearly three-fold more (841 DAPs), with 197 proteins shared between the two (Figure 4A). The higher number of DAPs detected in dormant seeds indicates greater proteomic variation across developmental stages relative to non-dormant seeds.

The temporal pattern of protein profiling showed that the number of proteins that increased in abundance declined in Phase IV in comparison to stages I–III (Figure 4B). Additionally, dormant seeds exhibited more phase-dependent specialization, with unique protein counts of 53, 149, 92, and 138 within Phases I–IV, respectively (Figure 4C). These data indicate that dormant seeds maintained higher proportions of DAPs in Phases II and IV (Supplementary Tables S1 and S2), supporting their importance as key developmental transitions for dormancy establishment.

### 3.3. Functional Analysis of DAPs

**Figure 5:**
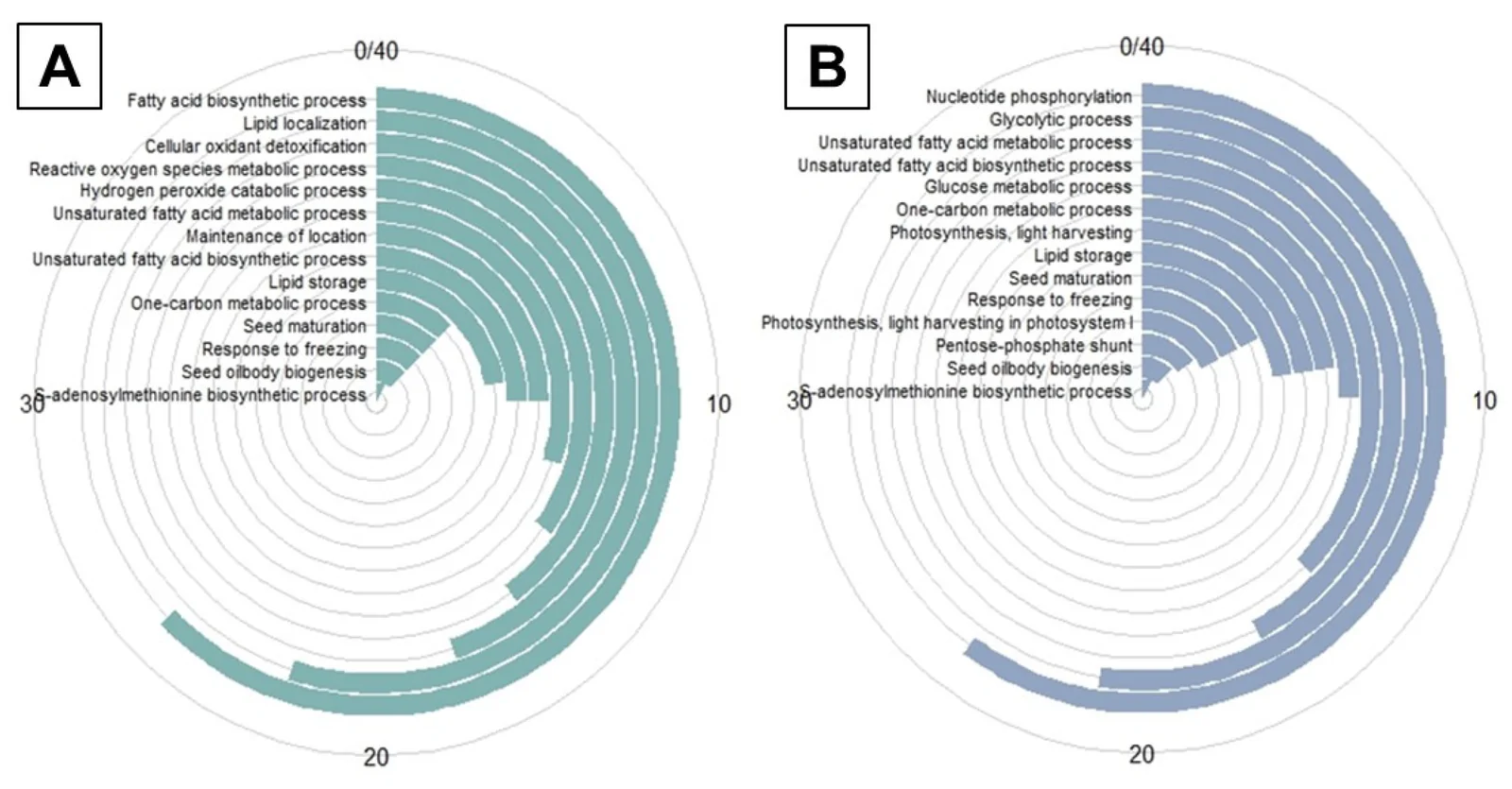
Functional profiling reveals a shift in biological priorities between dormant and non-dormant seeds. Comparative GO enrichment analysis of DAPs between (**A**) non-dormant and (**B**) dormant seed proteomes (arc size = term strength).

GO enrichment analyses were performed on all DAPs identified in non-dormant and dormant seeds, with results provided in Supplementary Tables S3 and S4, respectively. Based on this analysis, both seeds shared key enriched biological processes, including unsaturated fatty acid biosynthesis and metabolism, lipid storage, oil-body biogenesis, S-adenosylmethionine (SAM) biosynthesis, seed maturation, and response to freezing stress, highlighting their fundamental roles in *X. strumarium* seed development (Figure 5). Fatty acid biosynthesis, lipid localization, maintenance of localization, cellular oxidant detoxification, ROS metabolic process, and H2O2 catabolism were among the strongest GO terms in non-dormant seeds (Figure 5A and Supplementary Table S3). In contrast, the most prominent biological processes in the dormant seeds were nucleotide phosphorylation, glycolytic process, glucose metabolism, one-carbon metabolic process, photosynthesis, and pentose phosphate shunt (Figure 5B and Supplementary Table S4).

We subsequently performed independent GO enrichment analyses for each phase. The GO analysis detected a total of 84 and 490 pathways associated with developmental phases in non-dormant and dormant seeds, respectively (Supplementary Tables S5 and S6). GO terms identified in non-dormant seeds were largely shared with dormant seeds, with a limited number of exceptions, including plant vacuole and stress response pathways in Phase III and vitamin binding, IRE1-UPR, and lipid binding pathways in Phase IV (Supplementary Table S5). The following sections present the stage-specific GO terms, revealing how proteomic reprogramming differs temporally between dormant and non-dormant seed development.

#### 3.3.1. Phase I: Early Development

**Figure 6:**
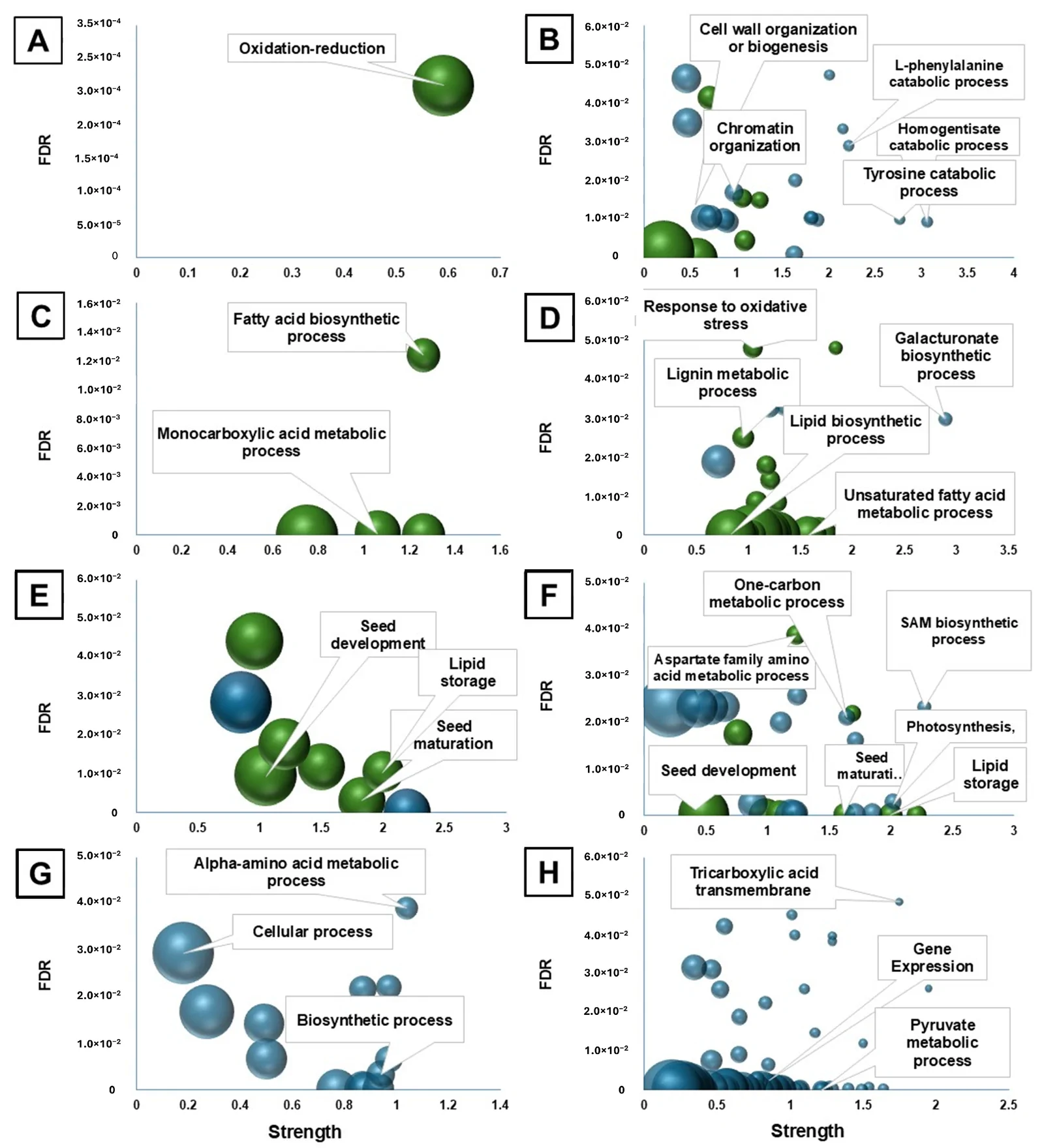
Functional enrichment analysis across seed development. GO term enrichment analysis in non-dormant (**A**,**C**,**E**,**G**) and dormant (**B**,**D**,**F**,**H**) seeds in different developmental phases (I–IV). Each graph displays the ‘metabolic view’ containing top-mapped pathways arranged by *p*-values (FDR) on the *y*-axis and the pathway impact/strength on the *x*-axis. Each green bubble indicates GO terms enriched by overrepresented proteins, while blue bubbles denote those enriched by underrepresented proteins. Bubble size reflects the relative abundance of proteins associated with each GO term.

GO analyses of over-represented (Up) and under-represented (Down) proteins at each developmental phase are included in Supplementary Table S7. During Phase I, both dormant and non-dormant seeds showed enrichment of oxidation-reduction-related processes associated with active metabolic activity during early seed development (Figure 6A and Supplementary Table S7).

Nevertheless, dormant seeds displayed additional enrichment of proteins associated with cell wall organization and biogenesis, chromatin organization, and catabolism of phenylalanine, tyrosine, and homogentisate (Figure 6B). Proteins associated with cell wall organization, including pectin-related modifiers and glycosyltransferases, were more abundant in dormant seeds (Table 1 and Table S7). Increased representation of proteins involved in H_2_O_2_ catabolism and phenol-containing compound metabolism was also detected (Table 1, Tables S2 and S7).

Dormant seeds, however, showed suppression of key enzymes involved in tyrosine catabolism and enzymes involved in ATP- and NADH-related metabolic processes, including GAPC2, GNAT, invertase, PHSL1, ATCEL2, and IMPL1. This collectively indicates a diminished capacity for energy production in the dormant seeds (Supplementary Table S2). Reduced abundance was also observed for several chromatin-associated proteins, including structural proteins, histone modifiers, replication-related factors, and transcriptional regulators (Table 1). Similarly, diminished levels of eIF5A, DRM1/ARP, DAP2, PHB3, and CYP83B1, which are involved in auxin signaling and metabolic control, were observed during this phase (Supplementary Table S2).

#### 3.3.2. Phase II: Mid-Development

Non-dormant seeds activated oxidation-reduction processes, monocarboxylic acids, and fatty acid metabolism in this phase (Figure 6C and Supplementary Table S5). In contrast, dormant seeds showed significantly enriched GOs related to ROS detoxification, unsaturated fatty acid biosynthesis, phenylpropanoid biosynthesis, lipid biosynthesis, fatty acid beta-oxidation, and lignin metabolic mechanisms, while proteins involved in galacturonate biosynthesis and pectin metabolism were significantly decreased in abundance (Figure 6D and Supplementary Table S6).

Dormant seeds exhibited overrepresentation of detoxification enzymes such as those involved in ROS metabolism, glutathione metabolism, flavonoid biosynthesis, and phenylpropanoid biosynthesis (Table 1). They further showed increased lignin biosynthesis, yet suppressed the pectin metabolic process (Table 1, Tables S6 and S7). Similarly, proteins involved in cellular lipid metabolic processes were significantly increased in abundance in dormant seeds, including those involved in lipid binding, biosynthesis, modification, and localization (Table 1 and Table S6).

#### 3.3.3. Phase III: Late Development

In the late developmental stage, both seed types displayed a reduction in the biosynthesis of unsaturated fatty acids, concomitant with increased lipid storage and localization, and activation of seed maturation and development pathways (Figure 6E,F; Table 1 and Table S7). Shared proteins associated with seed development included USPL1, OLEOs, seed storage proteins, and seed maturation proteins (Table 1 and Table S7).

Dormant seeds also exhibited marked suppression of photosynthetic activity, one-carbon metabolism, and SAM production, while metabolic flux through the aspartate-derived amino acid pathway was significantly elevated (Figure 6F; Table 1 and Table S7). The reduced representation of photosynthesis-associated GO terms is driven by diminished abundance of chlorophyll a–b binding proteins, ribulose bisphosphate carboxylases and PSI and PSII subunits.

#### 3.3.4. Phase IV: Maturation

Proteomic profiling during the maturation phase further uncovered divergent survival strategies between dormant and non-dormant seeds. Non-dormant seeds exhibited pronounced enrichment of abiotic stress response pathways (ABA, cold, and temperature; Supplementary Table S7), which is a signature classically associated with desiccation tolerance and maturation [38]. Our results also demonstrated systemic suppression in non-dormant counterparts of enzymes involved in cellular processes, biosynthesis, and amino acid metabolic processes (Figure 6G; Table 1 and Table S7).

In contrast, dormant seeds orchestrated a global quiescence program, suppressing processes such as tricarboxylic acid transmembrane transport, pyruvate metabolism, and gene expression-related processes (Figure 6H). Energy metabolism-related pathways were downregulated in mature dormant seeds, including respiration, glycolysis, and pentose phosphate, along with biosynthetic pathways for ATP, amino acids, lipids, carbohydrates, ribonucleotides, and sulfur/nitrogen/phosphate compounds (Table 1 and Table S7).

Other pathways significantly underrepresented in mature dormant seeds included those associated with gene expression machinery, including ribosomal proteins, ribonucleotide metabolism, elongation and initiation factors, amino acid biosynthesis, protein folding, and proteasome components (Table 1 and Table S7).

### 3.4. Gene Set Enrichment Analysis

**Figure 7:**
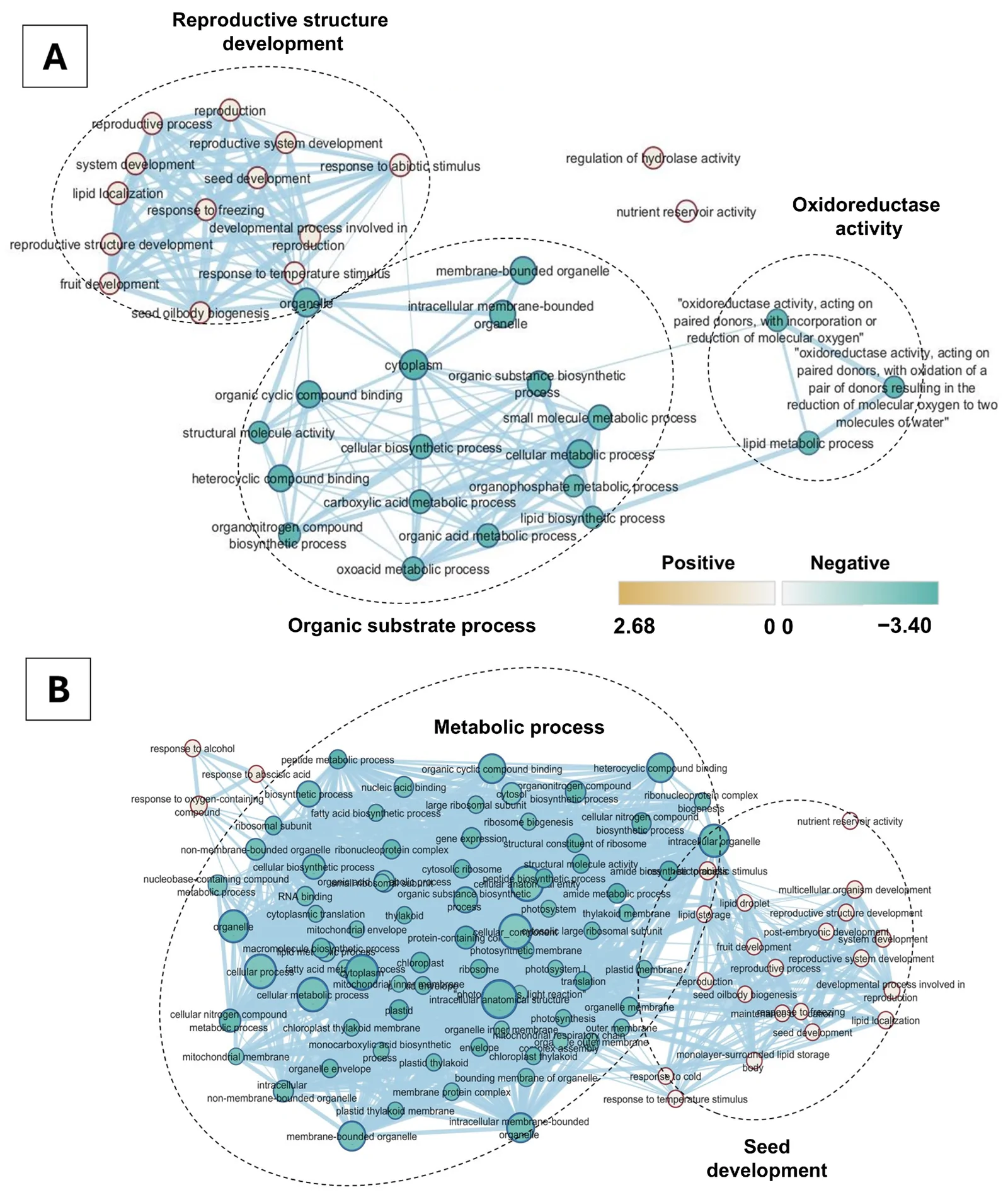
Functional network analysis of enriched metabolic pathways in *X. strumarium* seeds. Networks display interconnected pathways for (**A**) non-dormant and (**B**) dormant seeds. Dotted lines indicate predicted functional interactions between pathways. Each node (circle) represents a metabolic pathway, the size of which is dependent on the relative abundance of proteins associated with each pathway. The different colors (from blue to brown) represent negatively and positively enriched pathways, respectively.

Gene set enrichment analysis (GSEA) was performed as a complementary approach to identify coordinated pathway-level changes across proteins with moderate abundance differences. The results revealed conserved activation of seed developmental pathways in both seed types, with proteomic profiling demonstrating distinct specializations (Figure 7).

Non-dormant seeds predominantly showed an increase in the abundance of proteins associated with seed structure development, including several oleosin isoforms (OLEO1, OLEO6, A0A251URD4, A0A251RWY6, A0A251S7K5, A0A251U5P5), along with hydrolases and nutrient reservoir activities (Figure 7A and Supplementary Table S7). However, proteins associated with oxidoreductase activity, metabolism of small molecules, organophosphates, organic acids, oxoacids, carboxylic acids, and biosynthesis-related processes (organic substances, lipids, and organonitrogen compounds) were less represented in non-dormant seeds (Figure 7A).

In contrast, dormant seeds displayed a more intricate regulatory profile, characterized by a reduction in various metabolic processes, such as RNA binding, translation, thylakoid membrane functions, mitochondrial envelope processes, photosynthesis, cellular nitrogen compound synthesis, amide biosynthesis, and others (Figure 7B). Dormant seeds, however, showed increased representation of proteins involved in responses to cold, temperature, alcohol, ABA, and oxygen-containing compounds. Additionally, there was an increased representation of seed developmental processes, including lipid droplet formation, oil body biogenesis, lipid localization, nutrient reservoir activity, post-embryonic development, and other processes (Figure 7B).

**Figure 8:**
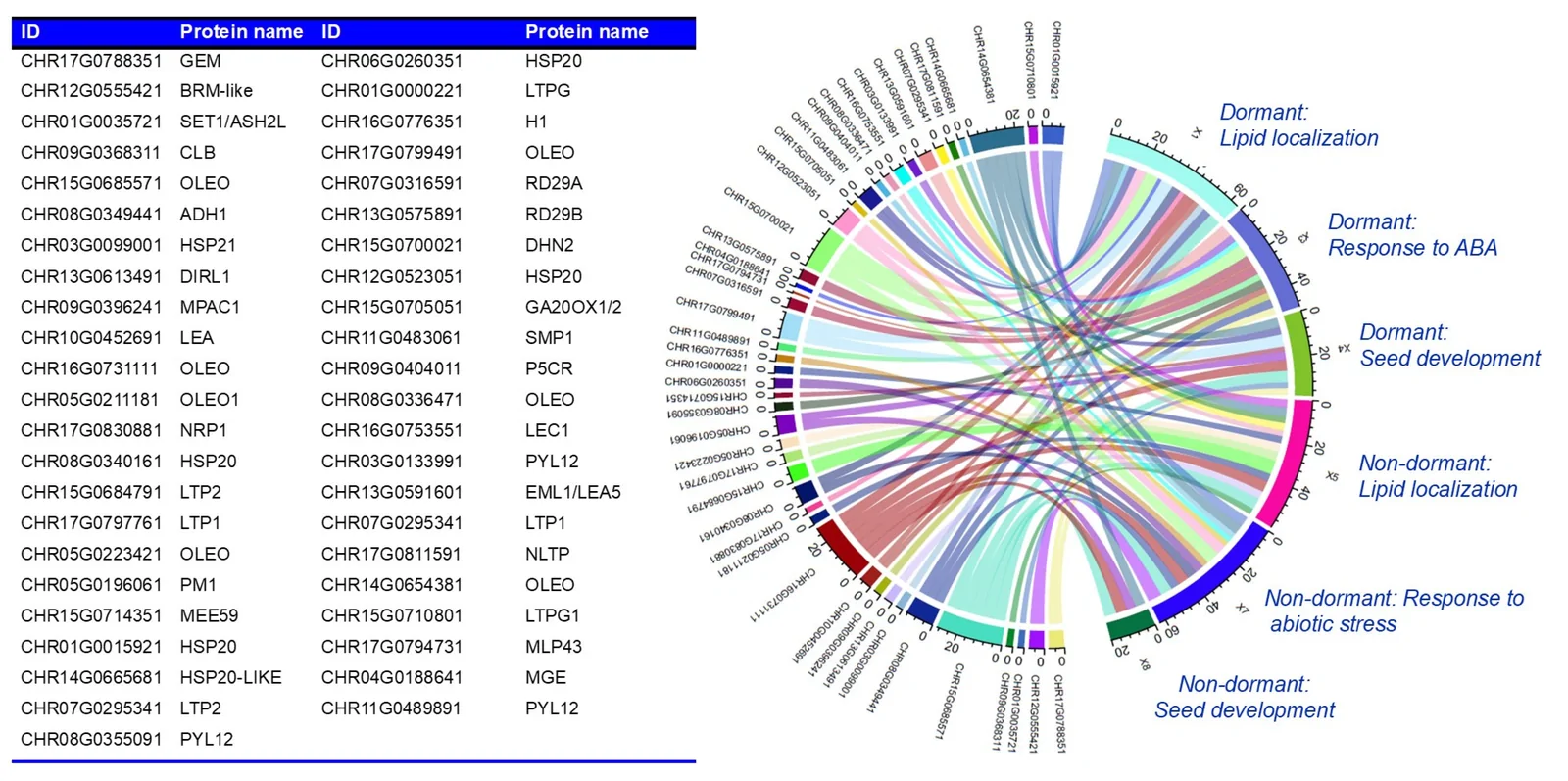
Network of key regulatory proteins and their biological functions during dormancy. Chord diagram illustrating core proteins associated with significantly enriched pathways in non-dormant (ND) and dormant (D) seeds of *X. strumarium* across four developmental phases. Pathway annotations (**right**) are connected to their respective core proteins (**left**) via arcs, with line thickness representing log_2_FC values. Connections indicate protein inclusion in each pathway’s leading-edge subset. Pathways are annotated numerically; for full names, see the table (**left**).

Pathway annotations further revealed that oleosins, lipid-transfer proteins (LTP1, LTP2, LTPG, DIR1L, NLTP) and dormancy-related components were among the core proteins that were differentially abundant in dormant and non-dormant seeds (Figure 8). The dormancy factors included chromatin remodelers (MEE59, H1, SET1/ASH2L), hormonal signaling pathways (FIP1, MLP43, different variants of PYL12, ERECTA/ERL, GA20OX2), stress-responsive proteins (ADH1, HSP20, HSP21, LEAs, DHN2, P5CR, RD29A, RD29B, EML1/LEA5, DRM1/ARP, SAG24, and MGE), and gene expression-related proteins (BRM, NF-YB6/LEC1, AGO4).

## 4. Discussion

### 4.1. Energy Limitation and Coordinated Suppression of the Cell Cycle Actively Constrain Early Growth in Dormant Seeds

The observed correlation between seed size and dormancy status in *X. strumarium* supports the hypothesis that resource allocation modulates seed size and germination behavior. Similar associations between seed size, reserve allocation, and dormancy intensity have also been reported in other plant species [39,40].

Early-developing dormant seeds coordinately suppress energy metabolism through multi-level pathways, including decreased abundance of tyrosine catabolism proteins and key enzymes for carbohydrate degradation, likely reducing TCA cycle substrates [41,42]. Instead, the activation of the pentose phosphate pathway, via increased abundance of G6PDH, supports a shift toward NADPH production and redox balancing rather than ATP-generating metabolism [43]. This highlights that non-dormant seeds probably have a higher ATP/NADPH ratio than dormant seeds.

The decreased abundance of cell cycle and auxin signaling regulators in dormant seeds further indicates a quiescent state with limited cell division. Auxin-dependent regulation of cell expansion and proliferation has previously been linked to embryo growth and seed developmental progression [44]. These changes suggest the idea that dormant seeds may prioritize embryo patterning and endosperm cellularization over active proliferation, consistent with previous findings of suppressed endoreduplication as a mechanism for cell size control [26]. This can mechanistically explain the observed size reduction in dormant counterparts.

### 4.2. Trade-Offs Between Pectin and Lignin in Dormancy Progression

The increased abundance of pectin-related proteins in early development aligns with studies showing that pectin modulates cell wall flexibility and hydration status, both of which are critical for seed coat formation and embryo development [45]. In contrast, the shift from pectin-dominated to lignin-dominated cell wall remodeling in mid-development can reflect a pronounced transition to secondary wall formation, a process critical for inducing dormancy in legumes and other species [46,47]. Such changes are often associated with seed coat strengthening and have been linked to physical dormancy mechanisms in several species [48]. Although physical dormancy was not directly assessed in the present study, the coordinated changes observed in cell wall-associated proteins raise the possibility that structural modifications of the seed coat contribute to dormancy establishment alongside hormonal and DOG1-mediated regulatory mechanisms [49].

Interestingly, the abundance profiling of GASA proteins showed a significant increase in early development and a decrease in the mid-developmental stages of dormant seeds. The GASA proteins integrate GA, ABA, and glucose signaling pathways during seed development, regulating seed germination and dormancy [13]. The proposed mechanism of action involves promoting cell wall remodeling, potentially through the mediation of expansins and hormone-mediated growth regulation. GASA also interacts with DOG1-LIKE proteins in an ABA/GA-dependent manner [50]. These findings point to the GASA family being an important component within the complex hormonal and molecular network associated with cell wall remodeling and dormancy-related processes during mid-development.

### 4.3. Lipid Metabolic Dynamics and Dormancy Regulation

The observed increase in the abundance of enzymes involved in unsaturated fatty acid and oleosin biosynthesis in both dormant and non-dormant seeds suggests a critical metabolic adaptation linked to membrane stability, energy storage, and stress response at the mid-developmental phase [51]. Similarly, the conserved core set of proteins expressed in late seed development of the two seed types shows that dormant seeds maintain maturation programs similar to those of non-dormant seeds, highlighting a shared developmental identity between seed types [52].

Nevertheless, the two seed types displayed a clear difference in lipid metabolism patterns in early and mid-developmental phases. The pronounced increase in the abundance of enzymes involved in unsaturated fatty acid synthesis can increase the production of polyunsaturated fatty acids (PUFAs) in dormant seeds, which serve two critical, interrelated functions: maintaining membrane fluidity [53] and providing precursors for lipid-derived signals like jasmonic acid (JA) [54]. Because JA and ABA signaling pathways have previously been shown to interact during dormancy regulation [55], the observed increase in fatty acid biosynthesis may be consistent with enhanced hormonal crosstalk during dormancy establishment.

Nonspecific lipid transfer proteins (nsLTPs) facilitate lipid transport and are involved in processes such as seed maturation, seed size determination, and germination [56,57]. In developing dormant seeds during phases II and IV, the reduction in several nsLTPs may also enforce dormancy by altering ABA homeostasis. Supporting this, an OsLTPL23 rice knockout mutant showed delayed germination [58]. Transcript profiles exhibited an increased abundance of ABA biosynthesis and responsive genes and downregulated ABA catabolism genes in mutants compared to wild type.

These findings can therefore suggest that fatty acid metabolism and lipid transport pathways may contribute to dormancy-associated developmental differences, potentially through effects on membrane properties and hormone-related signaling. Further experimental validation would be required to establish direct mechanistic links in *X. strumarium*.

### 4.4. Photosynthetic Suppression and Metabolic Quiescence in Dormant Seeds During Late Development

The marked decrease in the abundance of several photosynthesis-associated components during late development of dormant seeds indicates a negative effect on the photosynthetic apparatus. This suppression can minimize internal oxygen production and cellular respiration [59], alter seed metabolism and germination, and ultimately determine germination timing [60].

The reduction in the abundance of photosynthesis proteins in dormant seeds may limit transient sugar availability to the developing embryo, which cannot perform photosynthesis in vivo [61]. This may force the dormant seed into a state of profound metabolic quiescence essential for long-term viability and energy conservation. In this case, resources are probably redirected toward storage compound accumulation and stress-protectant synthesis, supported by the simultaneous increased abundance of pathways in dormant seeds related to seed development, lipid storage, seed oil body biogenesis, oxidation-reduction processes, response to stress, and aspartate family amino acid metabolism.

The increase in the abundance of the aspartate-derived amino acid pathway has previously been associated with nitrogen storage and redox balance during periods of reduced photosynthetic activity [62]. This pattern coincided with reduced abundance of GLNA2/GS2, the chloroplastic glutamine synthetase isoform responsible for assimilating NH_4_^+^ generated during nitrate reduction and photorespiration. Without photosynthesis, NH_4_^+^ assimilation is limited, leading to its accumulation. We hypothesize that maintaining GS2 activity during photosynthetic shutdown could be metabolically detrimental, a theory supported by findings in Arabidopsis *gln2* mutants, where GS2 activity, under conditions of reduced photosynthetic activity rather than NH_4_^+^ accumulation itself, was associated with toxicity [63]. Thus, the reduced abundance of GS2 in dormant seeds may reflect altered nitrogen metabolic requirements during dormancy-associated developmental arrest.

Conversely, non-dormant seeds sustain GS2 and other photosynthetic components at late developmental stages, which may support nitrate assimilation and nitrogen homeostasis during germination and early seedling establishment. Nitrate has previously been identified as an important germination-associated signal [64], and GS2 has been linked to NH_4_^+^ detoxification and osmolyte biosynthesis, including proline accumulation [65,66]. These contrasting molecular patterns may indicate distinct metabolic priorities between dormant and non-dormant seeds during late development.

It is worth noting that the increased abundance of glutamate-derived pathways in dormant seeds can channel nitrogen resources into stable storage compounds like asparagine [67]. The concurrent increased abundance of ALDH5F1, a succinic semialdehyde dehydrogenase, is thought to play an important role in increasing GABA content by reducing GABA degradation [68]. These patterns point to a nitrogen sequestration strategy through the accumulation of asparagine and GABA. Asparagine serves as a principal nitrogen transport and storage compound in developing seeds [69], while GABA represents a robust mechanism to maintain essential cellular pH homeostasis [70] and serves as a protectant during seed desiccation and heat stress [71,72]. GABA may further facilitate seed dormancy release via crosstalk between GA and ABA signaling pathways [73]. By accumulating these nitrogen-rich compounds, dormant seeds may ensure a ready supply of resources and signaling molecules essential for the transition from dormancy to germination once conditions become favorable.

### 4.5. Impaired One-Carbon-Sulfur Assimilation Characterizes Dormant Seeds in Late Development

The decreased abundance of proteins involved in one-carbon metabolism (e.g., tetrahydrofolate, Met, SAM) can directly impact downstream metabolic and epigenetic processes essential for germination in dormant seeds. Folate derivatives generated by SHM2 and FTHFS have previously been shown to contribute to nucleotide biosynthesis and support germination processes [74,75]. Folate plays a key role in synthesizing thymidylate and purines. Decreased abundance of these enzymes can deplete the nucleotide pool, which affects DNA replication and cell division, thereby resulting in seed dormancy [75].

The observed depletion of key sulfur assimilatory genes, such as SIR1 and MTND1/ARD, may restrict Met synthesis and consequently affect SAM availability, leading to reduced germination [76]. SAM serves as the universal methyl donor for DNA and histone methylation. Previous studies have shown that a deficiency in SAM biosynthesis can affect epigenetic regulation, including histone marks such as H3K9me2 [77,78]. These observations demonstrate that SAM is a dynamic regulator that integrates the metabolic status of the plant, specifically sulfur metabolism, with its epigenetic landscape. However, in the present study, epigenetic states were not directly measured, and therefore, such regulatory effects remain putative.

Nevertheless, the prominence of the Met synthesis protein MTO1/CGS in mature non-dormant seeds may reflect enhanced methionine biosynthetic capacity during late seed development. Overexpression studies in *Arabidopsis* have shown that MTO1/CGS activity can influence amino acid and carbohydrate accumulation, including methionine, sugars, and starch [79]. These metabolites have been associated with seed desiccation tolerance and germination potential [80]. Together, these observations suggest that sulfur and one-carbon metabolism may contribute to the metabolic distinction between dormant and non-dormant seeds during late developmental stages.

### 4.6. Carbon–Nitrogen Metabolic Imbalance at Maturation

Our GO analysis revealed a striking metabolic dichotomy during maturation. As expected, non-dormant seeds exhibited a strategic C-N reallocation toward stress acclimation. While they diminished nitrogen-intensive processes like amino acid metabolism and nucleobase-containing compound synthesis, they simultaneously increased abiotic stress-responsive pathways involving ABA, cold, and temperature stimuli. This can redirect carbon toward biosynthesis of osmo-protectants (proline) while minimizing nitrogen-dependent anabolism, reflecting a shift from active biosynthesis toward stress preparedness and maturation-associated physiology [81]. The reduction in nucleobase-containing small-molecule metabolism may further be consistent with decreased transcriptional activity during late developmental stages, as seeds present a quiescent state [82].

In dormant seeds, however, significant suppression of the glucose degradation, ATP biosynthesis, and pentose phosphate pathways minimized carbon flux through energy-yielding processes. The concurrent reduction in ribonucleotide metabolism and translational machinery restricted nitrogen investment into transcription and translation [83]. The suppression of various pathways, including those for ribosomal proteins, translation factors, amino acid biosynthesis, protein folding, and proteasome activity, contributes to a state of metabolic arrest for long-term soil persistence in dormant seeds [84].

Critically, the reduced abundance of proteasomal subunits in dormant seeds raises the possibility that protein turnover differs between dormant and non-dormant seeds. Central regulators of dormancy, such as the ABA-responsive transcription factor ABI5 and the GA signaling repressors DELLA proteins, are known substrates for proteasomal degradation [85]. Their accumulation, as a consequence of reduced proteasomal activity, would potently suppress the genetic programs required for germination. Proteasomal pathways have also been implicated in integrating ABA-GA-ET crosstalk during dormancy regulation. For example, group VII ethylene response factors are substrates of the E3 ubiquitin ligase PRT6, and the altered degradation of these proteins influences dormancy-related signaling and germination responses [14]. These findings provide a plausible framework for interpreting the reduced abundance of proteasome-associated proteins observed in mature dormant seeds, which may be associated with a dormancy-to-germination transition.

### 4.7. Temporally Distinct ABA Signaling Coordinates Early- and Late-Phase Dormancy Fate

Gene enrichment analysis indicated that while ABA-responsive proteins were globally reduced in abundance during seed development, their abundance patterns diverged significantly between dormant and non-dormant seeds. Key ABA signaling components were strongly increased in abundance in dormant seeds during both mid-development and maturation phases, but only transiently during maturation in non-dormant seeds.

Notably, NF-YB6/LEC1 was elevated in abundance during the mid-development phase of dormant seeds. LEC1 plays a dual role in morphogenesis and dormancy establishment by interacting with various TFs such as ABI3 and bZIP67 and is also known to regulate auxin-encoding genes [86] and DOG1 expression [87]. In addition to LEC1, MLPs, which share structural homology with ABA receptors such as RCAR/PYR/PYL [88], were also highly abundant in dormant seeds during mid-development. MLPs contain START-like domains that mediate ABA signaling through interactions with PP2C-SnRK2 complexes [89] and induce dormancy by modulating ABA/GA ratios [90]. PYL-mediated ABA signaling, a key regulator of dormancy establishment [91], was also increased during this stage. Concurrently, the decreased abundance of ERECTA/ERL proteins may contribute to enhanced ABA sensitivity in dormant seeds, consistent with studies where *erl* mutants show upregulation of dormancy inducers like ABI3, ABI5, and DOG1 [92].

These molecular signatures suggest that the mid-developmental phase may represent an important regulatory window for dormancy establishment, potentially involving DOG1-associated signaling networks. Nevertheless, DOG1 transcription can be enhanced by transcription elongation factor TFIIS/RDO2 [93]. The concurrent reduction in TFIIS abundance in dormant seeds may indicate altered transcriptional regulation during this phase. This may promote a conservative dormancy phenotype that influences DOG1 protein levels.

Analysis of the seed proteome at the late developmental stage (Phase III) revealed a molecular signature indicative of a signaling network for recalibration (Supplementary Table S2). This state is characterized by decreased abundance of the SnRK1 kinase KIN10 and the ABA receptor PYL12, whereas the phosphatases PP2C and PP2AA/PDF1 were increased in abundance. As the catalytic core of the SnRK1 kinase complex, KIN10 is a central activator of energy-consuming processes and a promoter of germination under favorable energy conditions, such as high glucose availability [94]. The reduction in KIN10 abundance may therefore suggest altered energy-associated signaling during late seed development in dormant seeds. This may contribute to ABA biosynthesis and signaling [95]. Further, the decreased abundance of PYL12 at the same time can lead to a weakened ABA perception and signaling cascade, potentially disrupting the delicate hormonal balance required to maintain dormancy [91].

DOG1 has also been reported to interact with the ABA signaling components. PP2AA/PDF1 functions as a negative regulator of seed dormancy upstream of DOG1 and may regulate DOG1 activity by promoting its dephosphorylation [96], which can influence DOG1 stability and its accumulation in dormant seeds. The elevated levels of PP2As can further enhance BR signaling pathways [97]. Therefore, the increased abundance of PP2A-associated proteins may contribute to broader hormonal signaling adjustments involving the ABA and BR pathways, which can potentially promote a recalibration of seed dormancy fate.

Similarly, PP2Cs interact with PYR/PYL/RCAR proteins, inhibiting ABA signaling and activating GA signaling, both of which promote the release of seed dormancy [98]. Other PP2C members (AHG1/3) interact with DOG1 in both dry and imbibed seeds [17,99]. DOG1 was shown to control seed dormancy by suppressing AHG1/3 activity, providing a convergence point for the ABA and DOG1 pathways. In the absence of DOG1 protein, AHG1 activity may be less constrained, potentially contributing to reduced SnRK2-mediated ABA signaling and a lower propensity for dormancy maintenance.

### 4.8. Chromatin-Based Modification Gates Dormancy Plasticity Across Seed Development

#### 4.8.1. Early–Mid Developmental Chromatin Remodeling Establishes Dormancy Competence

The reduction in the abundance of key regulatory proteins involved in chromatin remodeling (H1, H5, H2A, HMGA), histone modification (HUB1/RDO4, EMSY/ENT, HDs, MSI4/FVE, ATX1), and transcriptional activities (PAF1, RID1-like) suggests extensive chromatin-associated reprogramming during the early developmental stages of dormant seeds. These molecular signatures are consistent with coordinated developmental regulation that may contribute to dormancy establishment and seed developmental divergence.

The reduction in the abundance of chromatin remodelers can profoundly alter chromatin accessibility and compartmentalization, thereby determining seed dormancy and seed size. H2A acts as a chromatin-based switch for stress-response genes [100]. The loss-of-function mutants of the H2A isoforms showed increased sensitivity to ABA during seed germination via ABI3 [101]. These observations support a potential role for altered chromatin organization in ABA-associated dormancy regulation.

Interestingly, the decrease in the abundance of HMGA may represent an additional layer of gene regulation through epigenetic plasticity. A recent study on aging cells demonstrated that HMGA proteins serve as universal chromatin organizers that compartmentalize genes into functionally coherent 3D networks [102]. The reduced abundance of HMGA proteins observed in dormant seeds may therefore indicate altered chromatin organization during early development. This altered stabilization might reinforce transcriptional repression, thereby striking a balance between maintaining cellular uniformity and enabling the adaptive plasticity needed to respond to fluctuating environmental signals, although the downstream effects on chromatin states and transcriptional repression should be assessed in future studies.

PAF1C interacts with HUB1, a histone modifier, to facilitate chromatin remodeling [103]. Mutations in its components lead to dormancy induction [6]. By coordinating transcriptional elongation and histone modification, PAF1C creates a repressive chromatin environment that promotes dormancy induction through the regulation of genes like DOG1, NCED9, and CYP707A2 [104]. The reduced abundance of PAF1/HUB1-associated proteins observed here may therefore be consistent with altered transcriptional regulation during early dormancy establishment.

In addition to the PAF1C complex, the reduction in RID1, which encodes an ATP-dependent DEAD-box helicase, may disrupt the balance of RNA processing [105], thereby further stabilizing dormant states by inhibiting germination processes. This is consistent with the observed germination defects in *rid1* mutants, which mimic ABA hypersensitivity [106].

We further observed reduced levels of histone deacetylases (HD2A, HD2B, MSI4/FVE) and the reader EMSY-like, along with accumulation of the methyltransferase ATX1 in dormant seeds. HDAs function as transcriptional repressors by removing the histone mark H3K9/18ac, which is typically associated with active gene transcription. By removing these marks, HDAs may suppress genes involved in ABA catabolism, ET, and auxin signaling [107,108], thereby promoting a dormancy state. Interestingly, previous studies also reported that reduced HDA activity can increase acetylation at the DOG1 locus and promote DOG1 expression during seed development [109].

MSI4/FVE is a conserved subunit of polycomb repressive complex 2, which mediates gene silencing by histone H3K27 trimethylation. It has also been associated with dormancy-related pathways involving FLC [110] and GA biosynthesis, as well as *DOG1* regulation [111]. In parallel, the histone methyltransferase ATX1 has been shown to activate ABA-associated genes, including NCED3 [112], and to regulate secondary cell wall biosynthesis [113], linking epigenetic control to ABA signaling and structural changes. The reduced accumulation of EMSY-like proteins, which recognize the activating histone marks H3K4/36me3 [114], may additionally contribute to transcriptional regulation through chromatin-state recognition. The reduced abundance of EMSY-like proteins observed in dormant seeds may therefore be consistent with altered transcriptional accessibility during early–mid developmental stages.

Altogether, these observations suggest that chromatin-associated regulators may contribute to establishing a transcriptionally permissive or restrictive framework linked to dormancy-associated developmental programs in dormant seeds.

#### 4.8.2. Late Developmental Chromatin Reprogramming Enables Dormancy Plasticity

At maturation, a distinct epigenetic signature associated with the dormant state was also characterized by an increased abundance of the histone methyltransferases (ASH2R, SET1/ASH2, and SUVR4) and RNA helicases (RH23 and BRM-like), and a reduction in AGO4. These observations suggest dynamic chromatin-related regulation during late seed development.

The elevated SET1/ASH2 and ASH2R likely promote a permissive chromatin state at the FLC locus. The activation of the gene expression-associated proteins in developing seeds reinforced dormancy by modulating the hormonal balance towards higher ABA and lower GA levels [115]. Specifically, FLC influenced the ABA catabolic pathway and the GA biosynthetic pathway in a temperature-dependent manner. FLC is also known to repress FT [116]. Although we did not detect increased FLC abundance in dormant seeds, the observed FT reduction can be consistent with this established regulatory hierarchy. Transgenic Arabidopsis lines expressing FT driven by the 12S promoter exhibited significantly increased seed dormancy and earlier flowering via downregulation of GA biosynthesis and higher expression levels of DOG1 [111]. This evidence supports the idea that the suppression of the flowering signal FT may perturb the precise hormonal and genetic balance required for seed dormancy induction in response to environmental factors.

The increase in the abundance of SUVR4 presents an intriguing counterpart. Given that its homolog SUVH4 acts as a repressor of the dormancy genes DOG1 and ABI3 [5], the increased SUVR4 in dormant seeds may represent a compensatory mechanism or a preparatory signal for the transition to germination. In addition to the histone methyltransferases, the simultaneously elevated levels of the chromatin remodeler BRM can further contribute to the dormancy release through multiple pathways. BRM directly represses the expression of the dormancy promoter ABI5 [117] and activates GA biosynthesis [118]. BRM can also provide specific machinery to negatively regulate dormancy by influencing the DOG1 transcripts [119]. BRM binds to the 3′ end of the DOG1 locus and promotes the transcription of antisense DOG1 (asDOG1), a long non-coding antisense RNA derived from DOG1. This inhibits the translation of the sense DOG1 transcript in Arabidopsis. The suppression of DOG1 in dormant seeds with a higher abundance of BRM may further be responsible for the transition to germination along with hormonal signaling.

However, the reduction in AGO4, a key component of the RNA-directed DNA methylation pathway, highlights its role in post-transcriptional gene silencing to enforce dormancy. Evidence from wheat and Arabidopsis shows that reduced AGO4 expression is associated with a deeper dormant state [120,121]. Mechanistically, AGO4 primarily regulates genes involved in ET signaling and, to a lesser degree, in the regulation of ABA responses, with additional roles in integrating environmental signals to control dormancy [23,121]. These observations support a push-pull dynamic that can robustly enforce the dormant state while simultaneously priming the seed for its eventual transition to germination, ensuring a precise and adaptive response to environmental cues.

### 4.9. The DOG1 Transcript–Protein Disconnect Reveals an Environmentally Responsive Regulatory Layer

DOG1 is widely recognized as a master regulator of seed dormancy; however, its direct detection at the protein level is technically challenging [25,122]. In this study, we employed SWATH-MS, a state-of-the-art data-independent acquisition (DIA) method known for its extensive coverage, high quantitative accuracy, and ability to detect low-abundance proteins without the bias of intensity-based precursor selection [123]. Despite employing this sensitive and comprehensive approach, the DOG1 protein was not confidently identified in any of our proteomic samples. This absence invites two non-mutually exclusive interpretations, which we consider here.

First, from a technical perspective, the negative result may simply reflect limitations inherent to mass spectrometry-based proteomics [123]. The detection of any given protein depends on multiple factors including peptide ionization efficiency, the presence of uniquely mapping and readily ionizable tryptic peptides, sample complexity, and the depth of the spectral library used for identification [124]. DOG1, as a hydrophobic and low-abundance regulatory protein, may fall below the practical detection threshold of our current setup, regardless of the sensitivity of SWATH-MS. To critically evaluate whether the negative detection could be attributed to an intrinsic lack of suitable tryptic peptides for DOG1, we then performed an in silico tryptic digestion of the predicted DOG1 protein translated from the previously reported DOG1 transcript sequence [26]. This analysis predicted several peptides (length: 7–25 amino acids; mass range: 600–2800 Da) covering the entire DOG1 protein, including peptides with favourable ionization properties (Supplementary Table S8). Therefore, the absence of DOG1 detection in the present proteomic dataset may not be attributed solely to an intrinsic lack of suitable tryptic peptides. Nevertheless, this computational evidence does not exclude other technical limitations such as low abundance, ion suppression, or sample-specific degradation, which may still preclude detection despite the theoretical detectability of the protein.

Second, from a biological standpoint, the consistent observation of high DOG1 transcript levels (log_2_FC = 3.7)—derived from transcriptomic data in the same biological context [26]—reflects multilayered regulatory control operating between transcription and protein accumulation.

One possible explanation is that DOG1 transcripts are retained in developing seeds without being efficiently translated. The translation of DOG1 mRNA is known to be tightly reduced in fully matured dry seeds [125]. Instead of being immediately translated, the transcript is stored in dormant seeds, acting as a rapid-response mechanism that allows the seed to make a final germination decision based on immediate environmental cues [126]. Such regulation may provide a mechanism through which seeds remain responsive to environmental conditions encountered during maturation and subsequent imbibition.

Protein stability may provide an additional explanation. The stability of the DOG1 protein itself is exceptionally complex. It is well established that the expression of a single DOG1 splice variant is insufficient for protein accumulation and functional dormancy [8,122]. Instead, the co-expression of multiple isoforms is required to form stable protein complexes. Consequently, elevated transcript abundance does not necessarily result in detectable protein accumulation. Furthermore, several proteins identified in this study, including PP2AA/PDF1 and BRM, have been reported as negative regulators of DOG1 activity or accumulation. PP2AA/PDF1 reduces DOG1 stability through dephosphorylation [96], while BRM promotes *asDOG1* transcription, which suppresses DOG1 translation [119]. Their increased abundance in dormant seeds may hence contribute to limiting DOG1 protein accumulation despite the presence of DOG1 transcripts.

Environmental conditions during seed maturation may also influence this process. Temperature is known to affect dormancy depth [16] and has been associated with pathways linked to DOG1-mediated dormancy regulation [21,127,128]. Although cooler temperatures have been reported to enhance both DOG1 expression and protein accumulation, thereby promoting deeper dormancy [111,112], higher temperatures may have an opposing effect, limiting dormancy depth or preparing seeds for germination. Although our experimental design does not directly test temperature-dependent regulation of DOG1 translation or stability, the elevated temperature during maturation may have contributed to the observed disconnect between transcript and protein abundance. DOG1 has been proposed to function as part of a thermal-responsive regulatory network that can influence dormancy through changes in ABA sensitivity [127,129] and/or miRNA levels [9]. This interpretation is consistent with previous studies showing that maternal environment (e.g., temperature) during seed maturation can modify dormancy characteristics of offspring seeds through ABA-DOG1 interactions [127]. The persistence of DOG1 transcripts in the absence of detectable protein, therefore, suggests the presence of an environmentally responsive regulatory layer acting beyond transcriptional control.

Together, these observations suggest that DOG1-mediated dormancy regulation in *X. strumarium* may involve post-transcriptional and post-translational mechanisms that remain responsive to environmental conditions during seed maturation. Such regulation could provide flexibility in dormancy depth without requiring proportional changes in DOG1 transcript abundance. Further studies are needed to determine how environmental cues influence DOG1 translation, protein stability, and downstream dormancy-related pathways.

## 5. Conclusions

**Figure 9:**
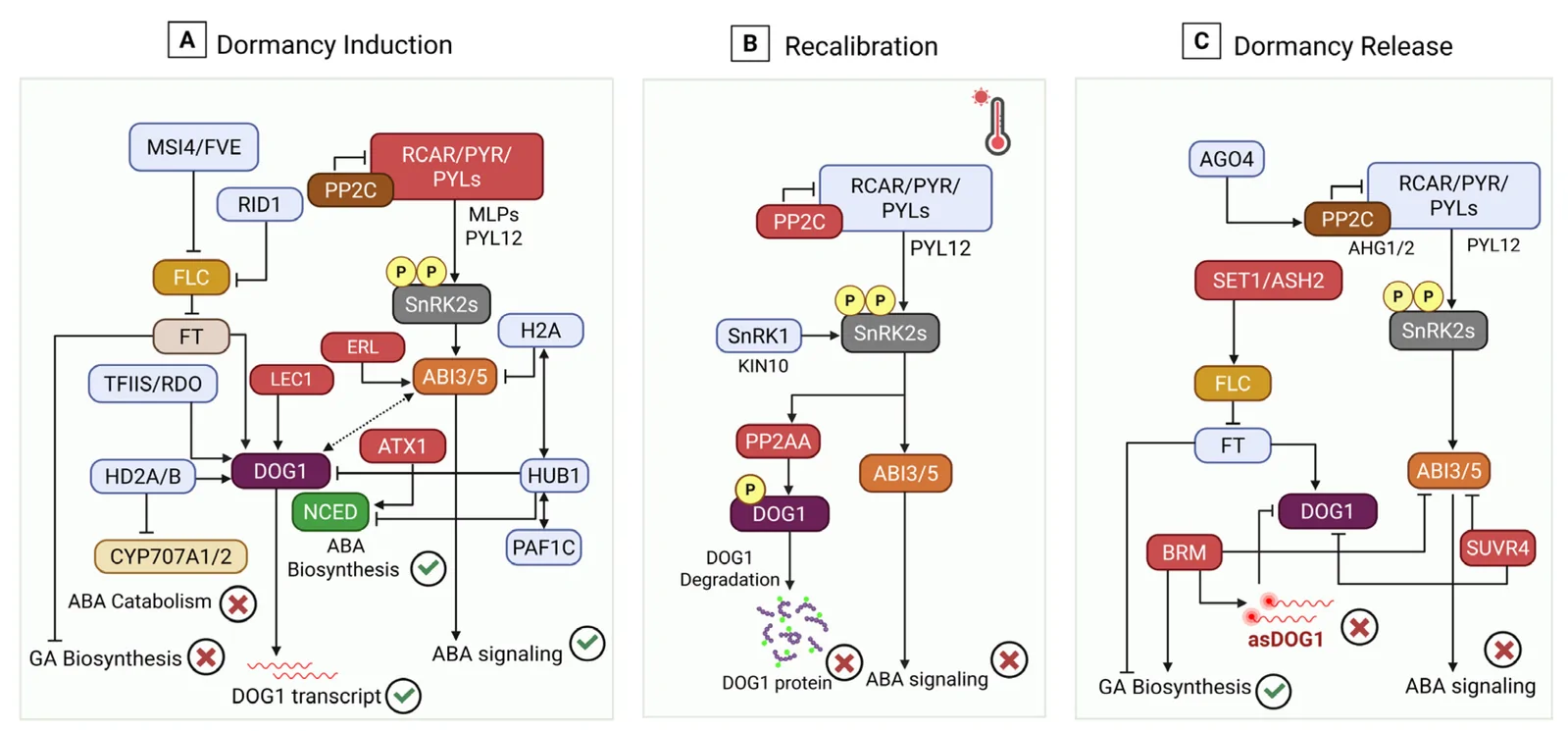
Schematic diagram of a two-phase mechanism for seed dormancy induction and release in *X. strumarium*, including early DOG1-dependent priming followed by an environmentally tunable phase at maturation. (**A**) Dormancy Induction: During mid-development, increased DOG1 transcript abundance, ABA-associated signaling, reduced ABA catabolism, and suppression of GA biosynthesis are associated with developmental processes favoring dormancy establishment. This phase is accompanied by broad metabolic and chromatin-associated regulatory changes. (**B**) Dormancy Recalibration: During late development, DOG1 transcripts remain abundant, whereas DOG1 protein accumulation may be post-transcriptionally regulated. Environmental factors, particularly maternal temperature, are proposed to influence dormancy depth through effects on DOG1-associated regulation and hormonal balance. (**C**) Dormancy plasticity and release potential: Changes in chromatin-associated regulators, hormone signaling, and *asDOG1*-related regulation may contribute to the adjustment of dormancy status during maturation, potentially enabling environmentally responsive dormancy release. Arrows: activation or positive regulation; blunt-ended lines: inhibition or suppression; dotted lines: indirect regulation; color-coded components: proteins increased in abundance (red), proteins decreased in abundance (blue). Proteins in other colors represent potential mediators in each pathway, without differentiation in abundance at the specific phase (created with BioRender).

Our integrative analysis of seed developmental proteomics, interpreted in the context of previously reported transcriptomic data [26], supports a two-phase framework for dormancy establishment in *X. strumarium*: an early DOG1-mediated priming phase followed by a temperature-sensitive plasticity phase during seed maturation. This two-phase model is presented as a working framework to guide future investigations in Figure 9.

During mid-development, elevated *DOG1* transcript abundance together with ABA-associated signaling and broad metabolic suppression were associated with a developmental trajectory favoring dormancy establishment. These changes coincided with reduced growth-related processes, altered cell wall remodeling, and coordinated shifts in energy metabolism, potentially contributing to the distinct developmental characteristics of dormant seeds.

At later developmental stages, dormancy regulation appeared to shift toward a more plastic state. Although DOG1 transcripts remained abundant, DOG1 protein was not detected, suggesting possible post-transcriptional or post-translational regulation (Figure 9B,C). Combined with changes in chromatin-associated regulators, hormone signaling, proteostasis, and metabolic pathways, these findings support a hypothesis in which dormancy depth may remain responsive to environmental conditions during seed maturation. In particular, maternal environmental factors such as temperature may influence this regulatory balance, although direct experimental validation will be required. These observations support a framework in which dormancy is better viewed as a dynamic and environmentally tunable developmental process rather than a fixed binary state.

Future studies should experimentally test the regulatory mechanisms proposed here. As a critical first step, we emphasize that the discordance between DOG1 transcript abundance and its undetectability at the protein level should be rigorously addressed using targeted proteomic validation approaches (e.g., qPCR, Western blot, PRM). Additionally, targeted analysis of DOG1 protein accumulation and splice variants, chromatin-state profiling (e.g., histone modifications and DNA methylation), metabolic validation (e.g., GABA, fatty acid composition, ABA/GA-related metabolites, etc.), and controlled maternal-environment experiments across temperature regimes will be important to evaluate the proposed framework. Because *X. strumarium* combines strong physiological dormancy with pronounced maternal responsiveness and natural seed dimorphism, it may serve as a valuable complementary system for investigating environmentally regulated dormancy mechanisms across species.

## 6. Limitations

Although this study provides valuable insight into mechanisms and processes involved in dormancy in *X. strumarium*, several limitations should be acknowledged. Post-translational modifications or specific proteoforms were not directly assessed because the proteomic analysis was focused on quantitation of total protein abundances. Such modifications may play critical roles in molecular signaling processes and warrant further investigation using top-down or targeted proteomic approaches. While this study allowed us to hypothesize a two-phase framework for dormancy establishment based on data analysis, it does not provide molecular evidence directly linking molecular changes to physiological outcomes. Future work should be aimed at providing experimental evidence to support the proposed novel dormancy mechanisms identified in this study.

## Figures and Tables

**Table 1:** Selected proteins and their corresponding functions identified in each GO term across the developmental phases of dormant seeds.

Phase	GO Term	Protein	Function
**I**	Cell wall organization and biogenesis	PAE1, PAE8, PME, PMEI, PMEI4, PbH1, PEL4, SBT1.5, and QRT3	pectin modifier
		ARAE2, FN3-like, GAUT9, and Exostosin (GT47)	glycosyltransferase
	Detoxification	GSTs, ATGSTU11, GPX, POXs, ADHs, ALL12, COMT1, GRP1, and RNS1	H_2_O_2_ catabolism and phenol-containing compound metabolism
	Tyrosine catabolism	FAH, HGO, and ADG6	
	Chromatin regulatory components	H1, H5, H2A, HMGA	structural proteins
		HUB1/RDO4, EMSY/ENT, HD2A, HD2B, MSI4/FVE, ATX1	histone modification
		PAF1, RID1-like	transcriptional regulator
		eIF5A, MCM7, and CENP-C/CNP3/MIF2	replication factors
**II**	Detoxification	POXs, CAT, SOD, APX1	ROS metabolism
		GST3, GPX, PHI2, GSTU6, GGT1	glutathione metabolism
		CFI1, U88B1, U88F3, CAMT, OMT1, IFRH, C81E1, ALL12/PRR1, FLS1, and CFI1	flavonoid biosynthesis
		PPO, 4CL2, CADH, COMT1, CAMT/CCoAOMT1, CADH1, oxidoreductase PILR2	phenylpropanoid biosynthesis
	Pectin metabolic process	PELs, PMEIs, BAHD acyltransferases, heme-dependent peroxidases, AIM1, CKX2, 3HCDH, GHD, AGAL1, GH3, BG14, GAUT9, GAE6, CCR1, laccase, and SNAK1/2	
	Lipid metabolic processes	MPAC1, MLP43, RNS1, DIR1, NBR1, AIM1, and LTP2/CDF3	lipid binding
		FAD2, FAD12, STAD, FADS-Likes, CYB5B, NCB5R/CBR1, ACP-like, CAC1, GDHRDHs, FABG1, KASI/II, KASIII, PKT3, PXG, and FabZ/FabA	lipid biosynthesis
		ACX3, AIM1, IMPL1, 3HCDH, MBOAT, FAO3, GDSL/SGNH, and PKT3	lipid modification
		NLTP2, DIR1, NLTP3, OLEO6	lipid localization
**III**	Seed development	LEGA, 11SBs, AT2S1, SESA1, 2S, PawL1, and 11SG	seed storage proteins
		SMP1, GLUB1, CRU2, PM1, and OBAP1	seed maturation proteins
	One-carbon metabolism	MTND1/ARD, GLYC1/SHM2, FTHFS, APS1, SIR1, APS1, and two variants of METK2/SAMS	
	Aspartate-derived amino acid pathway	ASP1, ASN3, and ALDH5F1	
	Photosynthesis	CB11, CB23, LHCA3, and LHCB1	chlorophyll a–b binding proteins
		RBCS and RBL	ribulose bisphosphate carboxylases
		PSAE, PSAF, PSAH, and	PSI subunits
		PSBO	PSII subunit
**IV**	Amino acid metabolic processes	TRA2, FBA1, GLN2, ASP1, ATAAT, MTO1, TPIS, ENO2, ASP5, ACP-like, SIR1, STAD, FABG1, CAC2, etc.	
	Metabolic-related pathways	ENO2, KPYA, FBA, GAPC-2, FBA, BAM2, MAOC, FBA1, FAHD2, ACLA-1, KPYA, and four components of pyruvate dehydrogenase complex	glycolysis
		TALs, FBAI, TKTs, TRA2, and 6PGDHC	pentose phosphate
	Gene expression machinery	RL2, RL4, RL6, RL9, RL15, RL18A, RL14b/L23e, RL22/L17, RL27, RL18a, RL28e, RL29, RUS2/RSSA, RUS5, RS8, RS16, RS19e, RS20, and etc.	ribosomal proteins
		EF1A, EF1D, EF1G, TIF3H1	elongation and initiation factors
		Biosynthesis of Leu (IIL1, IIL2, ATIMD2), Arg (ASS), Met (ARD/MTND1, SHM2, SAMS), Cys (CBS-like), ASP (AAT/ASP1), and branched chain amino acids (KARI-C, ACT, ASP1)	amino acid biosynthesis
		FKBP15-2, HSP70, HSP72, and chaperonins	protein folding
		RPT5A, RPT6A, MPN/RPN11/CSN5, TPP2, IMP1, ASPG1, and PSA3	proteasome components
		CAC2, NDK, NDPK, PURA	ribonucleotide metabolism

## Data Availability

The mass spectrometry proteomics data that support the findings of this study are openly available through the ProteomeXchange Consortium via the PRIDE database [130] with the dataset identifier PXD052504. Project Webpage: http://www.ebi.ac.uk/pride/archive/projects/PXD052504 (created 22 August 2024).

## Supplementary Materials

The following supporting information can be downloaded at: https://www.mdpi.com/article/10.3390/proteomes14030042/s1, Supplementary Figure S1: Canonical variate analysis (CVA) of the global proteomic profiles of *X. strumarium* seeds collected at 3, 10, 20, 30, and 45 days after burr emergence (stages 1–5, respectively). (A) Non-dormant seeds (*n* = 3). (B) Dormant seeds (*n* = 3). CVA was performed as a supervised multivariate analysis to visualize sample separation among developmental stages. The analysis showed close clustering of biological replicates within each developmental stage and was consistent with the stage-dependent proteomic patterns observed by PCA. Supplementary Table S1: Results of differential analysis in different developmental stages of non-dormant seed; Supplementary Table S2: Results of differential analysis in different developmental stages of dormant seeds; Supplementary Table S3: GO analysis using all identified DAPs in the non-dormant seed; Supplementary Table S4: GO analysis using all identified DAPs in the dormant seed; Supplementary Table S5: GO analysis using non-dormant seed DAPs at each phase separately; Supplementary Table S6: GO analysis using dormant seed DAPs at each phase separately; Supplementary Table S7: GO using over- and under-represented proteins (up and down, respectively) at each developmental phase. Supplementary Table S8: In silico tryptic digestion of the full-length DOG1 protein (160 aa) from *X. strumarium*.
