# Supplementary material for: DOG1-Mediated Priming Followed by Environmentally Tunable Plasticity: A Two-Phase Model for Dormancy Establishment in Xanthium strumarium
Source: Proteomes. 2026 Aug 21;14(3):42. doi: 10.3390/proteomes14030042 (PMC13511109; doi:10.3390/proteomes14030042)
Supplement: Supplementary file 1 [file proteomes-14-00042-s001.zip › NematiGholazidehetal_Supplementary Fig 1.pdf]

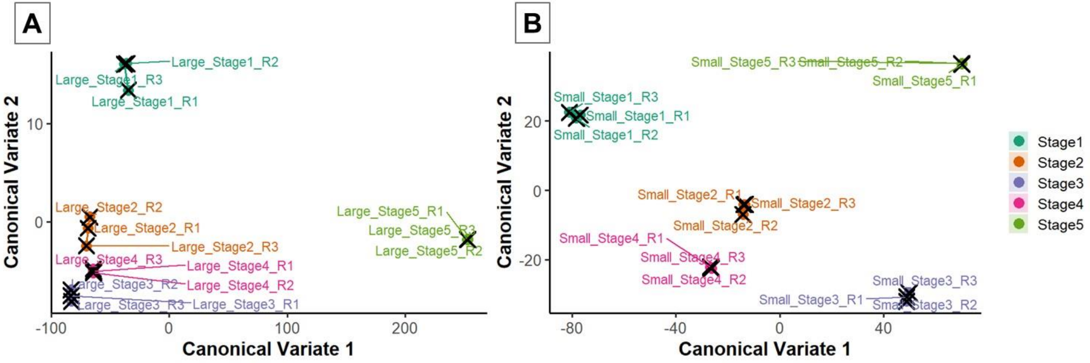

Supplementary Fig. 1 Canonical variate analysis (CVA) of the global proteomic profiles of *X. strumarium* seeds collected at 3, 10, 20, 30, and 45 days after burr emergence (stages 1-5, respectively). (**A**) Non-dormant seeds (n = 3). (**B**) Dormant seeds (n = 3). CVA was performed as a supervised multivariate analysis to visualize sample separation among developmental stages. The analysis showed close clustering of biological replicates within each developmental stage and was consistent with the stage-dependent proteomic patterns observed by PCA.
